# Enhanced microwave absorption performance of light weight N-doped carbon nanoparticles†

**DOI:** 10.1039/d0ra08455g

**Published:** 2021-02-17

**Authors:** Jianxin Chen, Peng Miao, E. Emily Lin, Ting Bai, Stoyan K. Smoukov, Jie Kong

**Affiliations:** MOE Key Laboratory of Materials Physics and Chemistry in Extraordinary Conditions, Shaanxi Key Laboratory of Macromolecular Science and Technology, School of Chemistry and Chemical Engineering, Northwestern Polytechnical University Xi'an 710072 China chenjianxin@nwpu.edu.cn kongjie@nwpu.edu.cn; Active and Intelligent Materials Lab., School of Engineering and Materials Science, Queen Mary University of London Mile End Road London E14NS UK

## Abstract

Microwave absorbents with specific morphology and structure have fundamental significance for tuning microwave absorption (MA). Herein, N-doped carbon sphere nanoparticles and hollow capsules were successfully fabricated *via* oxidative polymerization of dopamine in different mixed solutions, without any template preparation or etching process. Compared to solid particles, the microwave absorbents consisting of N-doped carbon with a hollow structure showed enormously enhanced MA performance, exhibiting a broad effective absorption bandwidth (from 12.7 GHz to 17.9 GHz) and a minimum reflection loss of −27.2 dB with a sample thickness of 2.0 mm. This work paves an attractive way for simple and eco-friendly preparation of advanced light weight microwave absorbents.

## Abstract 2

N-doped carbon particles were prepared through environmentally friendly and convenient methods. N-doped carbon capsules exhibit the best microwave absorption ability compared to their spherical counterparts.

## Graphical abstract

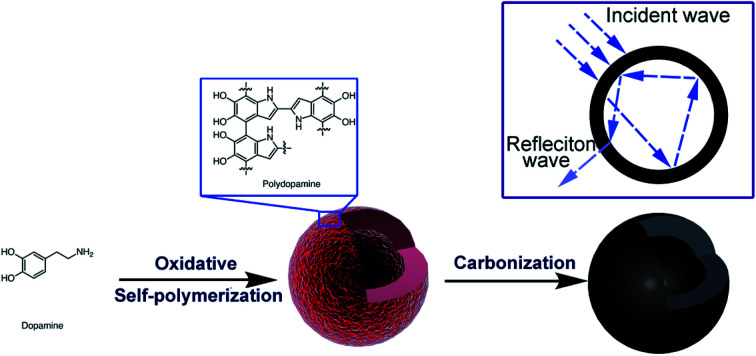

## Introduction

Microwave absorbents have attracted increasing attention because of their ability to safely convert incident electromagnetic energy into thermal energy,^2–5^ eliminating electromagnetic radiation and pollution which not only interfere with the normal operation of electronic devices, but also threaten the health of human beings. As is widely-accepted, an ideal electromagnetic absorbent should have the features of strong absorption, broadband absorption, light weight, low cost, excellent anti-oxidation capability and thermal stability simultaneously,^6^ which are required for practical application. Numerous microwave absorption materials (MAMs) have developed, including ferrites,^7,8^ metal alloys,^8,9^ conductive polymers^10,11^ or carbon materials,^5^ dielectric materials,^12–15^ metamaterials,^16,17^*etc.* Among them, carbon materials were regarded as an ideal candidate for their excellent electrical conductivity, light weight and great corrosion resistance.^18^

Besides composition, microstructure and geometric morphology also have a predominant effect on microwave absorption.^19,20^ Various carbon materials with different microstructure and geometric morphology, such as carbon core–shell nanoparticle,^21,22^ carbon hollow capsule,^23^ carbon nanofiber,^24^ carbon nanotube,^25–27^ porous carbon foam,^1,28^ and graphene^29,30^ have been reported as excellent microwave absorbents. Yin *et al.* reported the synthesis of mesoporous carbon hollow microspheres (PCHMs) with an interior void and a mesoporous shell. Microwave absorbents consist of PCHMs show a minimum reflection loss (RL) value of −84 dB at 8.2 GHz with a sample thickness of 3.9 mm.^31^ Han *et al.* improve the characteristic impedance and reflection loss of ordered mesoporous carbon through synergism of degree of graphitization and pore structure.^32^ The higher microwave absorption capability of carbon materials with porous or hollow nanostructure may due to their interfacial polarization and multi reflection loss. Meanwhile, this special structure usually has a lower density.

However, most of the porous or hollow carbon materials reported in previously literature were based on the carbonization of phenol/formaldehyde resin (RFR) after HF etching of the template.^33–36^ Developing microwave absorbents based on nontoxic carbon resources is still a challenging but desirable task. As a melanin-like biopolymer polydopamine is prepared from the polymerization of the nontoxic, widespread and sustainable bio-derivative, dopamine. Incorporating with lots of catechol, amine and imine functional groups, polydopamine can be readily deposited on virtually all types of substrates, including metals, metal oxides, polymers, and ceramics. Besides, polydopamine is also easy to chelating with many types of metal ions. Based on this striking property, polydopamine is, unsurprisingly, a very promising nontoxic carbon resources for heteroatoms doped or metal hybrid carbon materials.^37–40^ Li *et al.* prepared bowl-like carbon nanoparticles (BLCNs) through anisotropic self-assembly induced by emulsion interface. Microwave absorbents consist of BLCNs shown an enhanced MA performance which has a minimum RL of −45.3 dB and an effective absorption bandwidth (EAB) of 4.2 GHz.^41^

Herein, polydopamine nanospheres and capsules were successfully prepared by polymerization of dopamine in a water/ethanol or water/tetrahydrofuran mixture, without using any template. The resulting polydopamine nanospheres/capsules were subjected to two-step carbonization to produce carbon nanospheres/capsules with an integrated structure. Through this strategy, the carbon nanospheres/capsules with different sp^2^ carbon content were prepared and electroactive graphitic/pyridinic nitrogen elements were introduced into the matrix simultaneously. This makes the as-prepared carbon nanospheres/capsules highly suitable as microwave absorbents. The MA ability of as-prepared carbon nanospheres/capsules was tested in the region of 2.0–18.0 GHz. The N-doped carbon capsule composite exhibits a minimum RL of −27.2 dB at 14.8 GHz and the EAB of 5.5 GHz at a thickness of only 2.0 mm. The enhanced MA performance of capsule was observed and compared with that of solid carbon nanospheres. The main microwave attenuation mechanism of capsule composite could be attributed to the excellent impedance-matching and the multi-reflection derived from the hollow structure. Therefore, the as-prepared N-doped carbon capsule, prepared from nontoxic and sustainable bio-derivative through a facile process without template preparing and etching, provides vast flexibility for large-scale manufacturing of thin EMA materials with broadband microwave absorptions.

## Experimental

### Materials

Dopamine hydrochloride (98%) was purchased from Shanghai Macklin Biochemical Co., Ltd. Tetrahydrofuran (THF), ethanol and ammonia aqueous solution were purchased from Sinopharm Chemical Reagent Co., Ltd. Tris(hydroxymethyl) aminomethane was purchased from Aladdin Industrial Inc. All reagents are directly used without any further purification. Deionized water is used for all experiments.

### Synthesis of polydopamine spheres

Polydopamine (PDA) spheres were prepared according to the reported procedure.^42^ In detail, to a mixture of ethanol (80 mL) and deionized water (180 mL), different amounts of ammonia aqueous solution (NH_4_OH, 28–30%) was added under gentle stirring at room temperature for 10 min. Then, 20 mL dopamine hydrochloride aqueous solution (50 mg mL^−1^) was poured into the above solution under strong stirring. The reaction was carried out at room temperature for 48 h. Finally, after centrifugation the polydopamine spheres were collected and washed with deionized water for three times.

### Synthesis of polydopamine capsule

Polydopamine capsule was prepared according to the reported procedure.^43^ Typically, 200 mL THF and 300 mL Tris buffer (10 mM, pH = 8.0) were added into a flask with mild stirring for 30 min. Then 1.0 g dopamine hydrochloride was added into the mixed solution, and the reaction was carried out at room temperature for 7 days. After centrifugation the polydopamine capsules were collected and washed with deionized water for three times.

### Synthesis of N-doped carbon spheres and capsule

N-doped carbon spheres/capsule was prepared according to the reported procedure.^41^ Firstly, to a Teflon-lined stainless steel autoclave, the as-prepared polydopamine particles were added and re-dispersed in deionized water/ethanol mixed solution by ultrasonic. Then the autoclave was heating at 100 °C for 24 h to stabilize the nanostructure. Finally, N-doped carbon spheres/capsule were obtained by carbonization of the PDA spheres/capsules under the protection of inert nitrogen flow in a box type furnace. The heating rate was 5 °C min^−1^ up to the temperature of 800 °C. The carbonization of the precursor was carried out at 300 °C for 1 h and 800 °C for 3 h, under nitrogen flow. After that, the hollow capsule was spontaneous cooling to room temperature was performed keeping nitrogen flow.

### Characterization methods

Scanning electron microscopy (SEM, FEI Helios G4 CX) and transmission electron microscopy (TEM, FEI Talos F200X) were employed to characterization the morphology and structure of as-prepared samples. Element composition was analyzed by X-ray photoelectron spectroscopy (XPS) on a Kratos Axis Ultra DLD spectrometer, which employing a monochromated Al Kα X-ray source and a pass energy of 1486.6 eV (scanning voltage: 15 kV, scanning current: 10 mA). Raman spectra were collected with a WITec Alpha 300R confocal Raman microscope equipped with 532 nm laser excitation. Vector network analyzer (VNA, MS4644A, Anritsu, Atsugi, Japan) was employed to characterization the electromagnetic parameters of the N-doped carbon spheres/capsule (frequency range: 2–18 GHz). The mass ratio of as-prepared samples and paraffin is 2 : 1, and the samples were ultrasonically dispersed in the paraffin matrix at 60 °C for 2 h. The coaxial ring of samples with an outer diameter of 7.0 mm and inner diameter of 3.04 mm were prepared with tablet press for the test.

## Results and discussion

PDA spheres are synthesized *via* oxidative self-polymerization of dopamine in ethanol/water mixture solution. Reactions with different amounts of ammonia aqueous solution are performed according to Lu's strategy.^42^ The ratio of ammonia to dopamine was turned to adjust the size of PDA spheres. PDA spheres with different particle sizes (sphere 1: 80 nm, sphere 2: 200 nm and sphere 3: 440 nm) were prepared with different amounts of ammonia aqueous solution (5.0, 3.0 and 1.0 mL respectively). Carbon spheres prepared from carbonization of PDA spheres appeared to preserve the structural integrity and spherical morphology with a slightly decreased size.

PDA capsules are synthesized *via* the soft template method.^43^ THF is macroscopically miscible with water, but the macroscopically homogeneous THF–water mixtures are in fact phase-separated, as characterized by charge-transfer-to-solvent dynamics of tetrabutylammonium iodide^44^ and Laser Light-Scattering.^45^ The inhomogeneous domains in the THF–water mixtures solution could be utilized as a template of PDA capsules. As the oxidation and self-polymerization of dopamine occurring, more hydrophobic oligomers of dopamine separate out from the solution and aggregate on the interface to minimize the system energy, leading to the formation of the hollow structure.

**Fig. 1:**
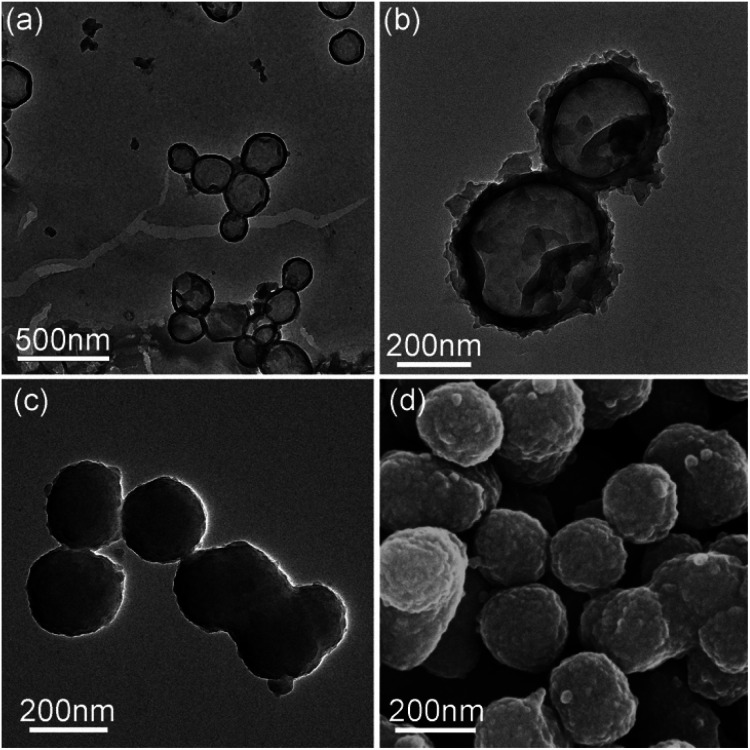
TEM graphs of (a) polydopamine capsule, (b) N-doped carbon capsule, (c) polydopamine sphere particle (sphere 2) and (d) SEM graphs of N-doped carbon particle (sphere 2).

The TEM images in Fig. 1 indicate that the PDA capsules possess the hollow sphere structure with a size of about 200–300 nm and the thickness of the shell was about 100. After carbonization under nitrogen atmosphere, the as-prepared N-doped carbon capsule showed no obvious change in the morphology, compared to PDA capsules. The surface of the capsules became rougher but the hollow structure still well-maintained.

**Fig. 2:**
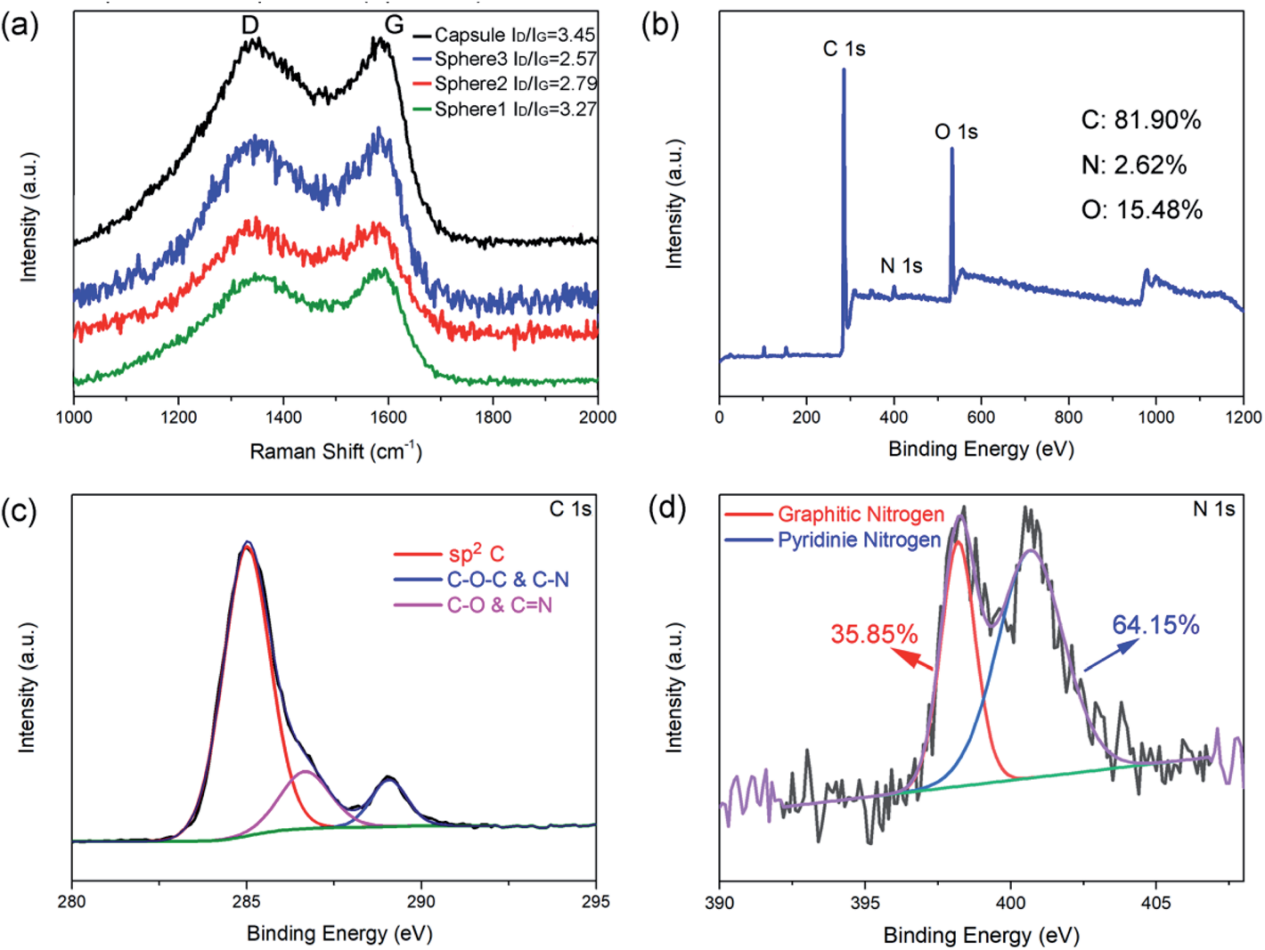
Raman spectrum of N-doped carbon particles (a), XPS survey spectrum (b), C 1s spectrum (c), and N 1s spectrum (d) of N-doped carbon capsule.

Raman spectroscopy and XPS were employed to investigate the structure and composition of the as-prepared N-doped carbon spheres/capsule. Fig. 2a shows the Raman spectra of four samples, all of which have two distinct peaks at around 1350 and 1580 cm^−1^. These two peaks are corresponding to D band and G band, respectively. It is generally accepted that D band is caused by the defects and disorder-induced features and G band is caused by the sp^2^ hybridized graphitic carbon.^46^ The characteristic D band and G band intensity ratio (*I*_D_/*I*_G_) is an indicator of the graphitization degree. By fitting the peak area with a Gaussian function, *I*_D_/*I*_G_ is calculated. *I*_D_/*I*_G_ value of the N-doped carbon spheres decreased with increasing particle size (3.27, 2.79 and 2.57 for 80 nm, 200 nm, and 440 nm, respectively). And the N-doped carbon capsule has the highest *I*_D_/*I*_G_ value of 3.45. It is reported that there is little sp^3^ C in the final carbon matrix derived from pyrolyzing of PDA at a temperature higher than 700 °C.^42^ In this article, particles undergo a pre-crosslink process (autoclave, 100 °C) before pyrolysis. O content in the as-prepared carbon particles (12–15%) is about twice higher than that without pre-crosslink (5%).^42^ The difference is attributed to the introduction of defects by the oxidation of surface.^47,48^ Small size and hollow structure are usually accompanied by a larger surface area, which may introduce more surface defects under oxidation. The co-existence of dipolar polarization and interfacial polarization were also confirmed by the *ε*′–*ε*′′curve (Fig. S7†). According to the Debye relaxation theory, one Cole–Cole semicircle represents one.

Debye relaxation process. All four samples shown several distinguishable semicircles in the measured frequency, implying the existence of multiple dipolar polarization. And the distorting of those semicircles indicating the existence of other mechanisms, such as interfacial polarization or Maxwell–Wagner effect.^14,49^

In addition, XPS analyses were employed to investigate the component of N-doped carbon particles obtained at different conditions (Fig. S4–S6†). Fig. 2b shows the XPS survey spectra of the N-doped carbon capsule and Table S1† shows the relative atomic ratios of each element. These results indicate that the different synthesis process of the PDA sphere and capsule has no obvious effect on the composition of the final product. The chemical composition and degree of graphitization of the N-doped carbon particles were decided by the pyrolytic carbonization process. And we observed a slight increase of O contents with decreasing particle size, this may be caused by higher surface oxidation. The high-resolution profile of the C 1s peak of the N-doped carbon capsule in Fig. 2c demonstrates that the C 1s peak could be divided into three peaks, which are correspond to C–C/C=C (284.6 eV), C–O/C=N (286.5 eV) and C–O–C/C–N (289.2 eV), respectively. The high-resolution profile of N 1s peak of the N-doped carbon capsule was shown in Fig. 2d. The two N 1s peaks at 398.5 and 401.1 eV can been assigned to pyridinic N (N atoms connected with two C atom) and graphitic N (N atoms connected with three C atom), respectively. All those samples have similar pyridinic N content range from 31.34% to 35.85% for the same carbonization condition (Table S1†).

**Fig. 3:**
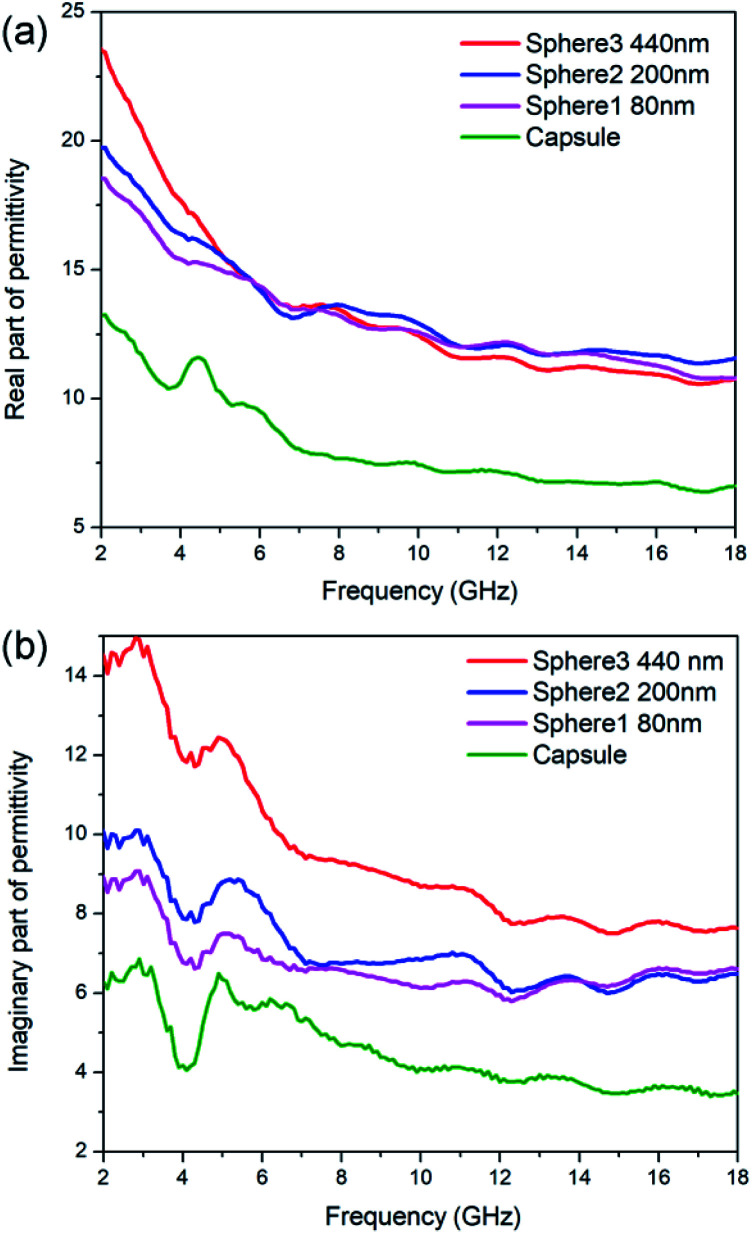
Permittivity for N-doped carbon particles. (a) Real parts, (b) imaginary parts.

Generally speaking, the microwave absorption of absorbents in media is chiefly depends on their complex permittivity (*ε*_r_) and permeability (*μ*_r_). In this work, the N-doped carbon particles have no magnetic loss, the microwave absorption performance mainly relies on dielectric loss ability. The complex permittivity is consist of two parts, the real part (*ε*′) and imaginary part (*ε*′′), where the *ε*′ represents the storage capability of microwave energy and the *ε*′′ represents the dissipation capability of microwave energy. The *ε*′ values of N-doped carbon sphere 1, sphere 2, sphere 3 and capsule are in the range of 18.52–10.80, 19.73–1.58, 23.51–10.77 and 8.40–4.49, respectively (Fig. 3). All the *ε*′ values of those samples decrease when the frequency is increased, as shown in Fig. 3a. The descending trend of the real part in the increased frequency of 2–18 GHz was caused by the dielectric relaxation effect, for the rotation of polarization cannot keep up with the change of external electric field in high frequency. Different to that of nanoparticles, the *ε*′ curves of nano-capsule show a peak around 4.5 GHz, this may be caused by the resonance behaviour due to the microwave multiple reflection in hollow structure. The morphology of carbon absorber also has a profound effect on its dielectric properties for their interfaces and multiple reflection. The accumulation of charge at the surfaces of membranes and caves increases the polarizability, especially at low frequencies.^50^ One typical example is the carbon hollow microspheres with uniform mesoporous shell (PCHMs). They show a multi peaks curve in *ε*′ curves.^51^ Hollow carbon fiber with a diameter of 1.10 μm also show a peak in low around frequency (7.0 GHz).^52^

The *ε*′′ values of sphere 1, sphere 2, sphere 3 and capsule are in the range of 8.89 to 6.61, 10.04 to 6.49, 14.48 to 7.64 and 6.27 to 3.46, respectively, as shown in Fig. 3b. The *ε*′′ values of those samples shown several obvious resonant peaks. This could be attributed to the dielectric polarization, including dipolar polarization and interface polarization, which is induced by the different electronegativity between the C atoms and doped heteroatoms.^53^ The similar resonance peaks in *ε*′′ curves in all particles were attributed to their similar dipole polarization originate from N-doping of carbon (pyridinic-N, graphitic-N) and defects. Nitrogen-doped Fe@C nanocapsules also show similar fluctuating curves while carbon materials without heteroatoms doping generally show a smooth curves.^54^ It is widely accepted that the *ε*_r_ of carbon materials is strongly dependent on two factors: the degree of graphitization and microstructure.^31^ It is proved by the aforementioned Raman spectra that the sample with the smaller particle size show a small degree of graphitization as subjected to the same pyrolysis process. But N-doped carbon sphere with small size still shows enhanced complex permittivity compared to the bigger one. This may be attributed to their special geometric morphology, as enhance of complex permittivity is found on particles with smaller sizes or hollow structure in the previous report.^23,52^

The RL value of absorbent can be calculated by the following formula deduced from the transmission line theory,^36^
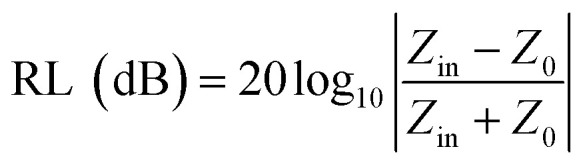

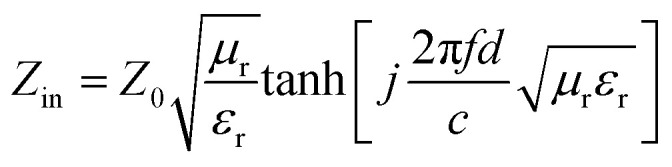
where Z_in_ is the input impedance at the air-absorber interface, *Z*_0_ represents the free space impedance. *μ*_r_ represents the relative complex permeability (*μ*_r_ = *μ*′ − *jμ*′′), in this work, *μ*′ ≈ 1, *μ*′′ ≈ 0, *ε*_r_ is the relative complex permittivity (*ε*_r_ = *ε*′ − *jε*′′). *c* is the speed of electromagnetic waves in vacuum condition, *d* is the thickness of absorbers and *f* represents the frequency.^55^ The RL value <−10 dB is generally selected as the criterion for effective absorption, which means > 90% electromagnetic wave was absorbed.

**Fig. 4:**
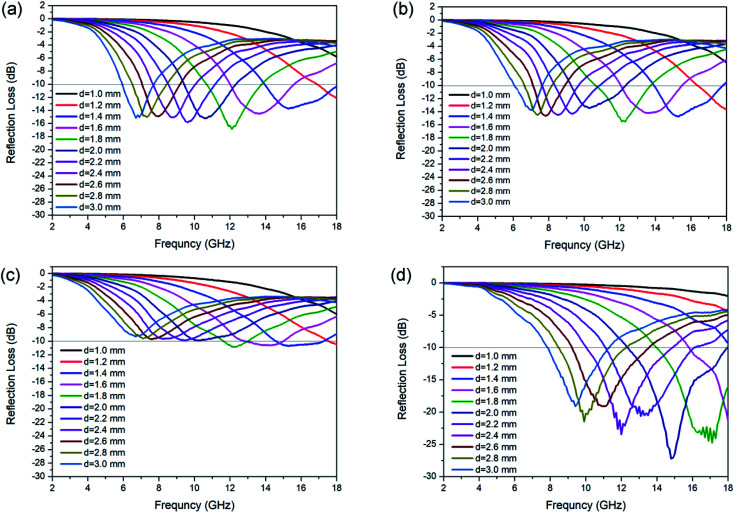
RL value of N-doped carbon particles, (a) sphere 1, (b) sphere 2 (c) sphere 3 and (d) capsule.

The calculated RL for each kind of N-doped carbon particles with various thicknesses is shown in Fig. 4. The maximum RL shifts to lower frequency for the geometrical effect (matching of thickness with frequency, *d* = *nλ*/4). For sphere samples with different sizes, there is an obvious enhance of RL performance with the decreasing of particle size from 440 nm to 200 nm, as the minimum RL are −10.9 and −15.5 dB at a thickness of 1.8 mm for sphere 3 (≈440 nm) and sphere 2 (≈200 nm) respectively. Further decreasing of particle size from 200 nm to 80 nm only shown a slight enhancement of absorption (RL = −16.8 dB). The minimum RL of sphere 1 (≈80 nm) can reach the widest EAB of 4.0 GHz at a thickness of 1.4 mm. N-doped capsules with hollow structure show the optimum enhanced microwave absorption with the minimum RL value of −27.2 dB at 14.8 GHz with a thickness of 2.0 mm, and the corresponding EAB can reach as wide as 5.5 GHz (12.7–17.9 GHz). We also characterized the microwave absorption performance of polydopamine particles. All of the three sphere particles shown poor microwave absorption ability, with a minimum RL value lower than −2.0 dB (Fig. S8†). Compared to sphere particles, polydopamine capsule shown a much better microwave absorption ability with a minimum RL of −16.6 dB (Fig. S8d†), which is close to that of N-doped carbon spheres. This result also illustrates the influence of morphology on electromagnetic wave absorption performance. But the EAB of polydopamine capsule is very narrow (8.3–9.2 GHz), which could be attribute to the lack of dipolar polarization. These results show that both morphology and element composition have an important influence on microwave absorption performance. Their synergy is essential for the design and synthesis of high-performance microwave absorbers. For further estimate the MA performance of N-doped carbon capsule, some typical carbon materials reported recently was listed in Table 1. Compared with those carbon materials, it can be found that the N-doped carbon capsule in this work exhibit comparable excellent EM wave absorption abilities. And it is worth to mention that our strategy provides a facile and convenient one-pot approach without any template etching process.

**Table 1:** Comparisons of MA performance of typical carbon materials between this work and previous works

Absorbents^a^	Thickness (mm)	RL (dB)	EAB (GHz)	Ref.
PCHMs-650	3.6	−39.4	3.49	36
HOPC	2.0	−17.4	4.5	57
RGO	1.8	−38.8	4.1	58
S-doped PCHMs	1.82	−51.83	6.08	6
NGNRs	3.0	−45	3.2	59
PCFs	3.0	−51	3.0	60
HCN-6	1.9	−50.8	4.8	23
BLCN	1.5	−45.3	4.2	41
N-doped carbon capsule	2.0	−27.2	5.5	This work

a PCHM: carbon hollow microspheres with a uniform mesoporous shell, HOPC: highly ordered porous carbon, RGO: reduced graphite oxide, S-doped PCHMs: sulfur-doped hollow carbon microspheres with mesoporous shell, NGNRs: nitrogen-doped graphene nanoribbons, PCFs: porous carbon nanofibers, HCN: hollow carbon nanosphere, BLCN: bowl-like carbon nanoparticle.

**Fig. 5:**
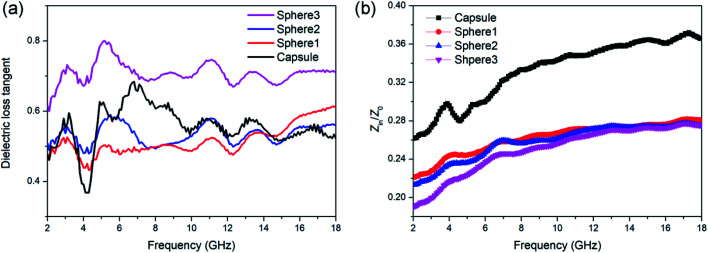
(a) Dielectric loss tangent (tan *δ*_*ε*_) value and (b) impedance matching (*Z*_in_/*Z*_0_) values of N-doped carbon particles.

For a better understanding of the different absorption abilities of the samples, the dielectric loss properties and impedance matching (*Z*_in_/*Z*_0_) were investigated in detail. The tangent value of dielectric loss (tan *δ*_*ε*_ = *ε*′′/*ε*′) could be used to evaluate the dielectric loss ability. Dielectric loss capabilities mainly originate from conductivity loss and polarization loss. As shown in Fig. 5, sphere 3 exhibit the best dielectric loss capability. This is mainly attributed to the conductivity loss for sphere 3 contains more graphite composition and thus better electrical conductivity. But sphere 3 didn't show the best MA ability, which may due to their poor matching-impedance. Too high permittivity might be detrimental to the impedance matching and results in vigorous microwave reflection, hinder the absorption.^56^ When evaluated at the same thickness of 2.0 mm, N-doped carbon capsule exhibit a distinct better impedance matching than sphere particles (Fig. 5b). And the hollow structure provides enhanced interfacial polarization and multiple reflection. The synergy of these factors resulted in the enhanced microwave absorbing properties of the as-prepared N-doped carbon capsule.

## Conclusions

In summary, sphere particle and hollow capsule were prepared through environmentally friendly and convenient methods by oxidative self-polymerization of dopamine, without any template preparation or etching process. After pyrolysis of polydopamine at 800 °C, N-doped carbon particles could be obtained with intact shapes and highly effective MA ability. N-doped carbon capsule exhibits the best MA compared to spherical counterparts. The minimum RL value of N-doped carbon capsule is −27.2 dB and the corresponding EAB is 5.5 GHz at 2.0 mm thickness. Our work gives insight into the environmentally friendly preparation of N-doped carbon materials as microwave absorbent, which has great potential in electronic devices and systems.

## Conflicts of interest

There are no conflicts to declare.

## Supplementary Material

RA-011-D0RA08455G-s001
