# Supplementary material for: Enhanced microwave absorption performance of light weight N-doped carbon nanoparticles
Source: RSC Adv. 2021 Feb 17;11(14):7954–60. doi: 10.1039/d0ra08455g (PMC8695094; doi:10.1039/d0ra08455g)
Supplement: RA-011-D0RA08455G-s001 [file RA-011-D0RA08455G-s001.pdf]

Electronic Supplementary Material (ESI) for RSC Advances.
This journal is © The Royal Society of Chemistry 2021

Enhanced microwave absorption performance of lightweight N-doped carbon nanoparticles

Jianxin Chen[1,2,*], Peng Miao[1], E Emily Lin[2], Ting Bai[1] Stoyan K. Smoukov[2] and Jie Kong[1,*]

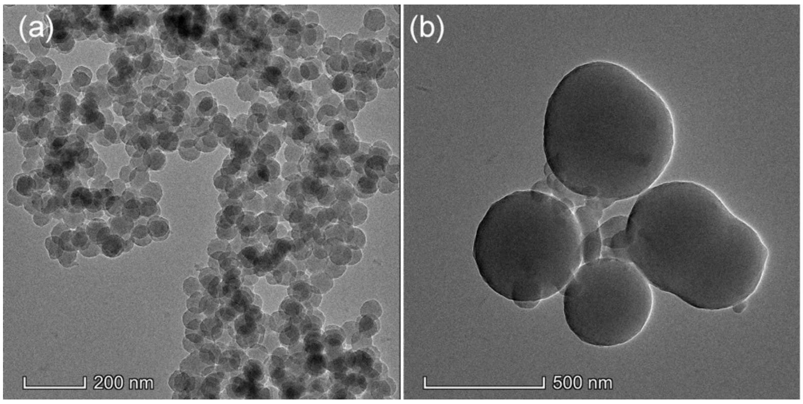

Figure S1 TEM graphs of polydopamine particle. (a) Sphere1, (b) Sphere3

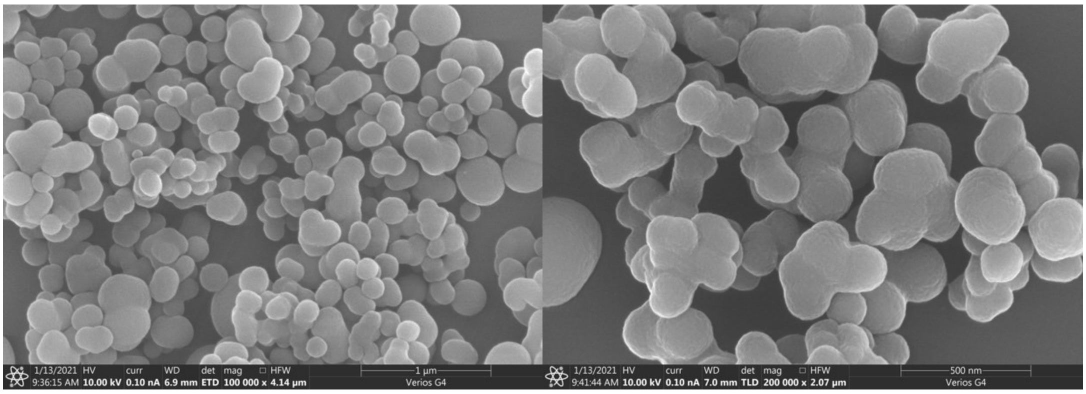

Figure S2 SEM graphs of N-doped carbon capsule.

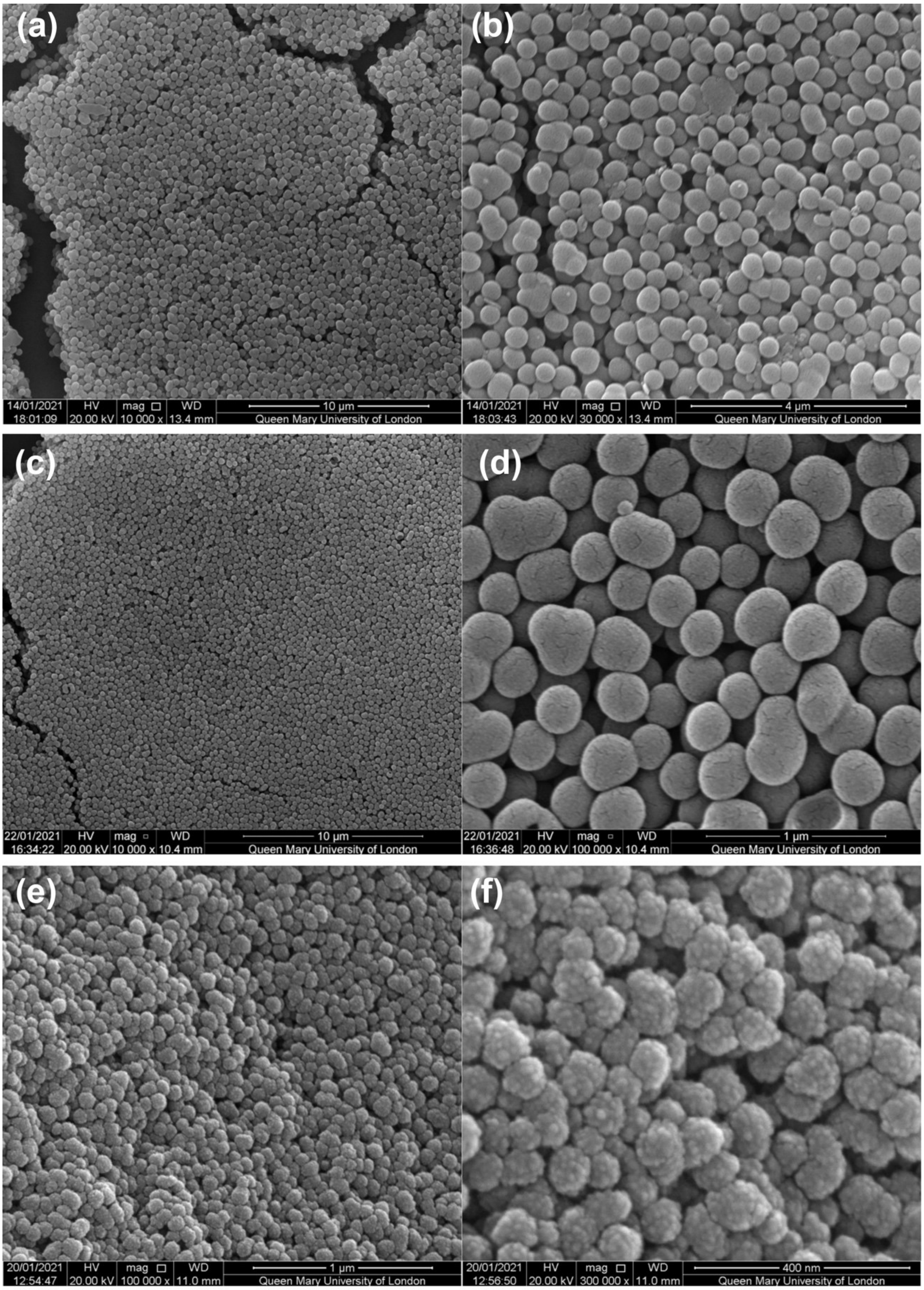

Figure S3 SEM graphs of N-doped carbon particle. (a, b) Sphere3, (c, d) Sphere2 (e, f) Sphere1

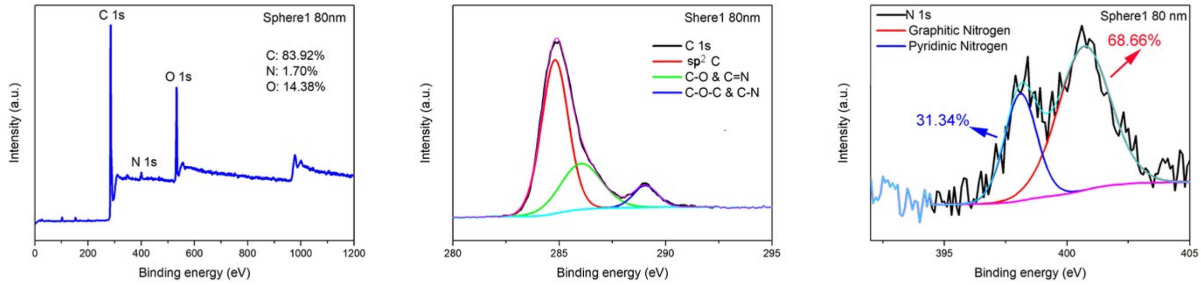

Figure S4 XPS spectrum of N-doped carbon particle (Sphere1).
(a) survey spectrum, (b) C 1s spectrum

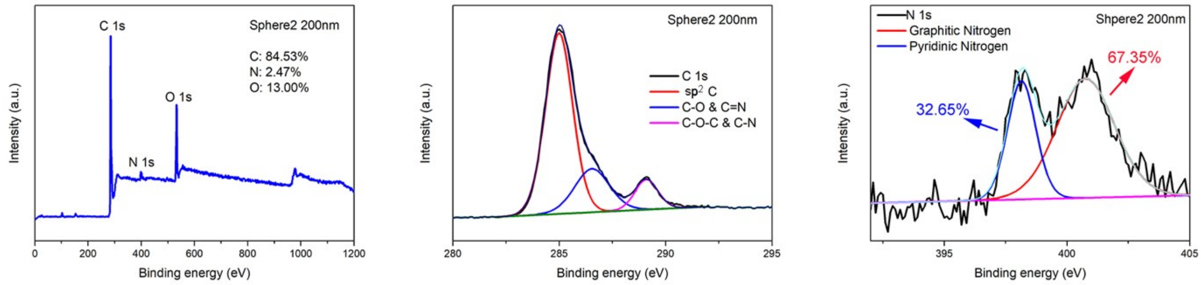

Figure S5 XPS spectrum of N-doped carbon particle (Sphere2).
(a) survey spectrum, (b) C 1s spectrum

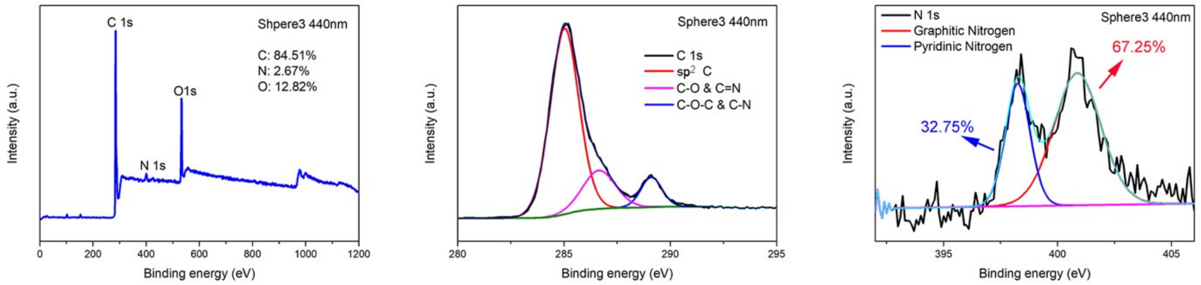

Figure S6 XPS spectrum of N-doped carbon particle (Sphere3).
(a) survey spectrum, (b) C 1s spectrum

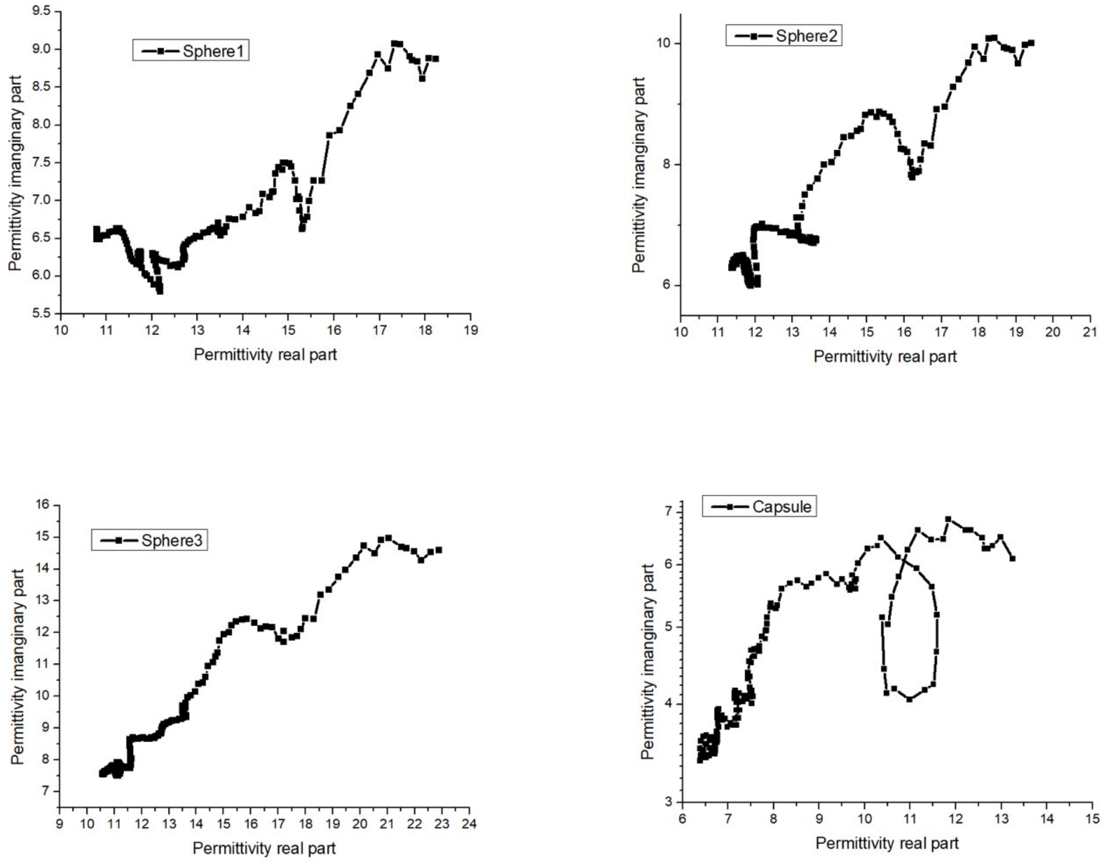

Figure S7 Cole-Cole semicircles of N-doped carbon particles.

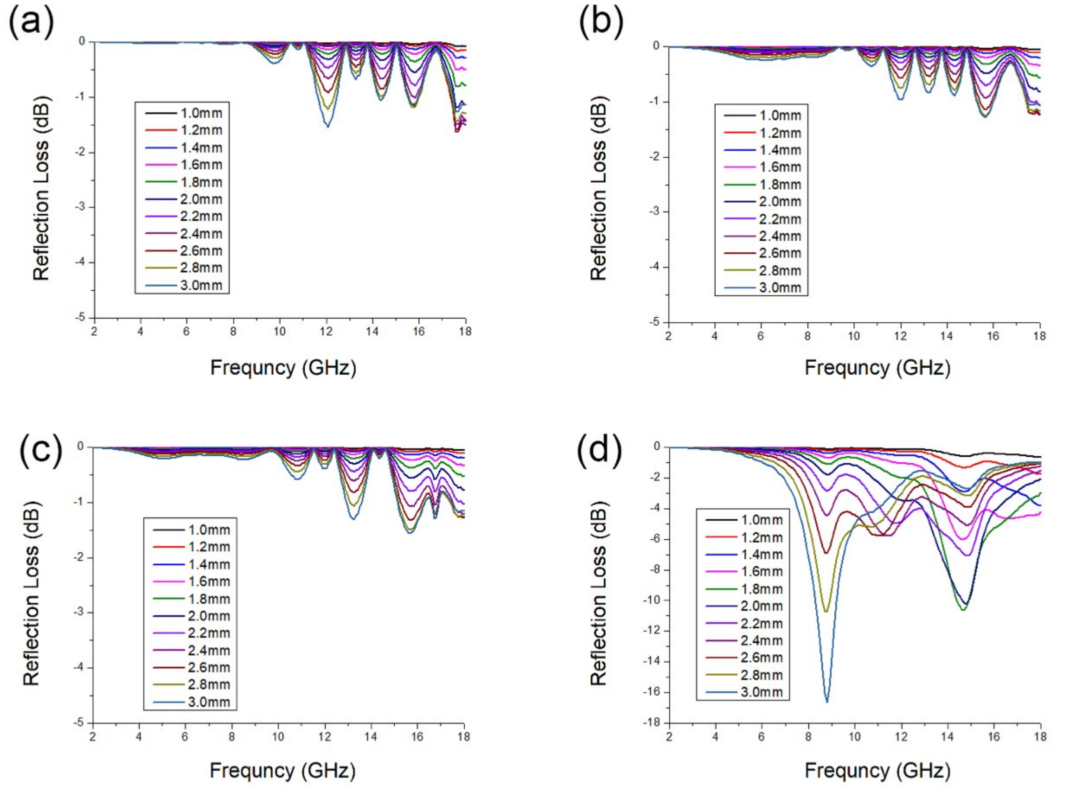

Figure S8 *RL* value of Polydopamine particles, (a) Sphere1, (b) Sphere2 (c) Sphere3 and (d) capsule.

Table S1 Elementary compositions of N-doped carbon spheres and capsules.

	Size	O(at%)	C(at%)	N(at%)	pyridinic N
**Capsule**	200-300nm	15.48	81.90	2.62	35.85%
**Sphere1**	80 nm	14.38	83.92	1.70	31.34%
**Sphere2**	200nm	13.00	84.53	2.47	34.67%
**Sphere3**	440nm	12.82	84.51	2.67	32.75%
